# Web-based surveillance of respiratory infection outbreaks: retrospective analysis of Italian COVID-19 epidemic waves using Google Trends

**DOI:** 10.3389/fpubh.2023.1141688

**Published:** 2023-05-18

**Authors:** Gloria Porcu, Yu Xi Chen, Andrea Stella Bonaugurio, Simone Villa, Leonardo Riva, Vincenzina Messina, Giorgio Bagarella, Mauro Maistrello, Olivia Leoni, Danilo Cereda, Fulvio Matone, Andrea Gori, Giovanni Corrao

**Affiliations:** ^1^Biostatistics, Epidemiology and Public Health Unit, Department of Statistics and Quantitative Methods, University of Milano-Bicocca, Milan, Italy; ^2^National Centre for Healthcare Research and Pharmacoepidemiology, University of Milano-Bicocca, Milan, Italy; ^3^Directorate General for Health, Lombardy Region, Milan, Italy; ^4^Centre for Multidisciplinary Research in Health Science, University of Milan, Milan, Italy; ^5^Department of Informatics, Systems and Communication, University of Milano-Bicocca, Milan, Italy; ^6^PoliS Lombardia, Milan, Italy; ^7^Agency for Health Protection of the Metropolitan Area of Milan, Lombardy Region, Milan, Italy; ^8^Local Health Unit of Melegnano and Martesana, Milan, Italy; ^9^ASST Fatebenefratelli-Sacco, Luigi Sacco Hospital – University of Milan, Milan, Italy; ^10^Department of Pathophysiology and Transplantation, School of Medicine and Surgery, University of Milan, Milan, Italy

**Keywords:** autoregressive moving average, COVID-19, Google Trends, epidemic wave, exponentially weighted moving average control chart, syndromic surveillance

## Abstract

**Introduction:**

Large-scale diagnostic testing has been proven insufficient to promptly monitor the spread of the Coronavirus disease 2019. Electronic resources may provide better insight into the early detection of epidemics. We aimed to retrospectively explore whether the Google search volume has been useful in detecting Severe Acute Respiratory Syndrome Coronavirus outbreaks early compared to the swab-based surveillance system.

**Methods:**

The Google Trends website was used by applying the research to three Italian regions (Lombardy, Marche, and Sicily), covering 16 million Italian citizens. An autoregressive-moving-average model was fitted, and residual charts were plotted to detect outliers in weekly searches of five keywords. Signals that occurred during periods labelled as free from epidemics were used to measure Positive Predictive Values and False Negative Rates in anticipating the epidemic wave occurrence.

**Results:**

Signals from “fever,” “cough,” and “sore throat” showed better performance than those from “loss of smell” and “loss of taste.” More than 80% of true epidemic waves were detected early by the occurrence of at least an outlier signal in Lombardy, although this implies a 20% false alarm signals. Performance was poorer for Sicily and Marche.

**Conclusion:**

Monitoring the volume of Google searches can be a valuable tool for early detection of respiratory infectious disease outbreaks, particularly in areas with high access to home internet. The inclusion of web-based syndromic keywords is promising as it could facilitate the containment of COVID-19 and perhaps other unknown infectious diseases in the future.

## 1. Introduction

Monitoring and accurate real-time surveillance of disease spread are essential to create situational awareness and initiate timely responses (1). During the current Coronavirus Disease 2019 (COVID-19) pandemic, population-level surveillance has relied primarily on aggregated results from individual laboratory testing (2). Most laboratories worldwide have reported considerable shortages in test kits, reagents, and qualified personnel required to perform the diagnostic testing for SARS-CoV-2 infection, leading to underestimations of the true epidemiological situation of COVID-19 and suggesting the need for alternative surveillance methods to anticipate outbreaks and the dynamics of the pandemic (3, 4).

Syndromic surveillance is an emerging approach in this field, defined as the ongoing systematic collection, analysis, and interpretation of “syndrome” specific data for early detection of public health threats (5). Syndromic surveillance systems seek to use existing data in real-time to provide immediate analysis and feedback to policymakers (6–8). Technologies using social media, search queries, and other internet resources are novel and inexpensive approaches for detecting and tracking emerging diseases. Such approaches, which constitute the new field of Infodemiology and Infoveillance (9), have been successfully used in the cases of SARS (10, 11), influenza (12–19), Ebola (20–22), and measles (23, 24), among others. During the COVID-19 pandemic, several studies have been conducted using web-based platforms where users self-report or search for their health-related issues. Search engines, particularly Google (1, 25–41), have been considered for COVID-19 surveillance purposes, highlighting their potential as complementary sources of information for population-level surveillance of pandemic spread. Previous studies using these data have yielded valuable lessons in their appropriate use, including avoiding non-specific search terms and ensuring suitable analyses (42).

It should be emphasised that infodemiology metrics are promising tools, especially in countries where most people actively use the Internet daily. Italy, Romania, and Slovenia are among the few European countries where less than half of the citizens use the Internet daily (43). In addition, the Italian Institute of Statistics (ISTAT) reports disparities in home Internet access during the pandemic period even within the Italian territory, between northern and southern regions (44, 45). Thus, since substantial differences in internet access are reported between Italian regions and the COVID-19 pandemic stroke Italy with varying intensities and periods, the study of the performance of tracking pandemics with infodemiologic metrics across Italy could be considered a natural experiment aimed to infer the functioning of this source in different conditions. Finally, as access to the internet changes over time, suitable models that can identify unexpected anomalous use of certain keywords while correcting for the natural variability of the process should be used (46). However, to the best of our knowledge, analytical tools, such as Autoregressive Moving Average (ARMA) models (47) and control charts (48), have never been used to model web-based data aimed at detecting early signals of COVID-19 outbreaks.

Autoregressive tools were applied to data from the most popular web-based platform (Google Trends) to verify whether unexpected anomalous use of certain keywords might detect SARS-CoV-2 infection outbreaks early with respect to surveillance systems based on nasopharyngeal swabs. Data from three regions located in the North (Lombardy), Centre (Marche), and South (Sicily) of Italy, where Internet access differs strongly, were used for the current application. A set of sensitivity analyses was performed to account for sources of systematic uncertainty in this study.

## 2. Methods

### 2.1. Catchment areas

This study used data from three Italian regions, including Lombardy (Northwest), Marche (Central), and Sicily (Southern Italy). The data covered more than 16 million citizens, nearly 28% of the Italian population.

### 2.2. Data sources

SARS-CoV-2 infections were ascertained according to real-time reverse transcription-polymerase chain reaction (RT-PCR) assay of nasopharyngeal swabs processed from a laboratory accredited by Regional Health Authorities. The date of confirmed diagnosis was the day swab processing was completed, and the patient tested positive. The weekly number of confirmed SARS-CoV-2 infections was used as a reference for evaluating and comparing syndromic data from web-based data sources.

Google Trends (49) was used to search for the weekly intensity using a set of non-specific COVID-19-related terms (i.e., syndromic respiratory concepts, which we will call “keywords” hereafter). The related five keywords, the Italian translations of “cough,” “fever,” “sore throat,” “loss of smell,” and “loss of taste,” were chosen according to those used by selected previous publications on this topic (1, 42, 50, 51). Google Trends does not provide absolute search numbers but instead provides a measure entitled interest over time that ranges from 0–100, with 0, 50, and 100 indicating that there is insufficient data for the term, “the term is half as popular,” and the term is at its peak popularity, respectively (29). For consistency, the values of the weekly searches were transformed to range from 0 to 100.

### 2.3. Statistical modelling

#### 2.3.1. Statistical Process Control (SPC) remarks

Google searches always occur over time, irrespective of the pandemic or its exacerbation. Nevertheless, searches are expected to increase whenever, and ideally before, an epidemic wave is reported from the swab-based surveillance system. Therefore, the amount of Google searches may be considered time series processes in which observations exhibit “natural” statistical variability (46). As a result of persistent random variability of the process and variations due to systematic and predictable reasons (e.g., Google search is expected to increase yearly, as well as to show a certain seasonal variability), the monitored process should be flagged as out-of-control whenever the observed value significantly exceeds that expected (7). The expected value is obtained taking into account the “natural” variability of the process (7). Alterations in the process characteristics result in variations in the observed values, resulting in more observations exceeding the control limits and the process being flagged as out-of-control. Distinct steps are required for developing the SPC procedure.

#### 2.3.2. Autoregressive Moving Average (ARMA)

The “natural” variability of the in-control data was captured and used to establish the in-control distribution. In our application, because the time process of interest forms a time series with seasonal variations, we used a regression model with ARMA (1,1) error terms (52) to fit the data during the control period preceding the onset of the pandemic in our geographic setting (i.e., from 2015 until 2019). The response variable, $y_{t}$ , denotes the amount or count in weeks t. Thus the general form of the regression model is $y_{t}=\mu_{y_{t}}+\varepsilon_{t}\;t=1,2,\ldots$ where $y_{t}$ is the mean response which is expected to be affected by a set of time-related predictors (e.g., season, month, year), and $\varepsilon_{t}$ is an error term that follows an ARMA (1,1) process $\epsilon_{t}=\phi_{1}\varepsilon_{t-1}+\beta_{t}-\theta_{1}\beta_{t-1}\;$ where $\phi_{1}$ and $\theta_{1}$ are the AR (AutoRegressive) and MA (Moving Average) coefficients, respectively.

Predictors’ effects were estimated for the dummy variables month-of-the-year, with M_1_ to M_11_ representing January to December (skipping July, which was used as the reference) (7). The sine and cosine functions are used for considering seasonal effects. In addition, the yearly-trend variable t was included. Therefore, the mean response was modelled as $\mu_{y_{t}}=\beta_{0}+\;\sum_{i=1}^{11}\beta_{i}M_{i}+\beta_{21}\sin\;\left(\frac{2\pi t}{12}\right)+\beta_{22}\cos\;\left(\frac{2\pi t}{12}\right)+\beta_{23}t$ . Residuals are the differences between observed and model-based expected values. Since residuals are depurated from seasonality and trends, they can be used to construct a control chart to monitor abnormal increases in the amount of Google searches.

#### 2.3.3. Exponentially Weighted Moving Average (EWMA) control chart

The actual monitoring starts by comparing the incoming data with the in-control distribution to determine whether and when the process goes out of control. The monitoring is commonly visualised using a control chart, where process scores are plotted against time. The EWMA procedure, introduced by Roberts (53) to detect mean changes across time, was used in this application. The procedure combines past and current information and tracks a weighted sum of the original observations, where more recent observations receive higher weights (54). At each measurement occasion of the actual monitoring period (i.e., for each week starting from 1 January 2020 to 31 December 2021, with i = 1,…,104), the exponentially weighted moving average z_i_ is calculated as $Z_{t}=\lambda \;x_{t}+\left(1-\lambda \right)\;z_{t-1}$ , where *x_t_* denotes the observation at each measurement occasion *t*. The starting value *z*_0_ is equal to the first step average $\mu^{\wedge 1}$ . The parameter 0 < *λ* ≤ 1 provides a weight applied to the current observation; lower values permit the detection of smaller mean changes. A *λ* value of 0.05 was used in the current application according to SPC literature recommending values between 0.05 and 0.25 (55). The control limits and central line of the EWMA chart are given by $\mu_{0}\pm L\sigma \sqrt{\left(\frac{\lambda }{2-\lambda }\right)\left[1-{\left(1-\lambda \right)}^{2t}\right]}$ , where μ_0_ is the centreline (56). The EWMA chart was generated in this way to identify possible outlier signals, defined here as any weekly observation falling outside the control limits of the EWMA control chart. The algorithm was applied using the dedicated functions of the ‘surveillance’ package in R (57).

### 2.4. Model performance

The weekly incidence rate of SARS-CoV-2 infections detected by the conventional surveillance system during the entire observation period (i.e., from January 2020 until December 2021) was plotted and compared with residuals of the weekly trends in Google searches during the same period. That is, syndromic proxies are expected to detect epidemic waves sooner. The timeliness of detection was assessed qualitatively by visual inspection of plots.

More analytically, the occurrence of modelled outliers (i.e., observed values exceeding the upper limit of the 95% confidence band of expected ones) was compared against the weekly swab-based alarms. We identified the “weeks consecutively affected by an epidemic wave” and, for exclusion, those “free from epidemics” from the first week of 2020 until the last week of 2021. The weeks consecutively affected by an epidemic wave started when, for the first time, the number of positive swabs in a given week increased by 10% that of the previous week, with the corresponding week denoting the “onset of an epidemic wave.” The wave ended when the weekly incidence rate of positive swabs returned to values lower than the average weekly rate of the considered semester. In addition, among the 104 weeks of interest, we denoted an “outlier week” as those affected by an outlier signal.

To investigate whether the occurrence of an outlier correctly predicts the onset of an epidemic wave, we computed the proportion of weeks labelled as “outlier weeks” that fell in a “free from epidemics” subperiod, which were followed within a given “time-lag” by the “onset of an epidemic wave.” This measure was denoted as the positive predictive value (PPV) of a significant outlier occurrence.

In addition, to assess whether the absence of an outlier falsely predicts the onset of an epidemic wave, we computed the proportion of weeks free from an outlier signal that fell in a “free from epidemics” subperiod, which were followed within a given “time-lag” by the “onset of an epidemic wave.” This measure was denoted as the false negative rate (FNR), defined as one minus the negative predictive value (NPV) of the absence of a significant outlier occurrence.

The discriminant performance represents the ability of an outlier to generate true signals (that is, early detection of the start of an epidemic wave while excluding false signals). The discriminant performance was assessed graphically by plotting the PPV against FNR for time lags ranging from 1–8 weeks.

Model performance was assessed separately for each of the five considered keywords (please see the section “Data Sources”) and for all the keywords together (i.e., a week was considered to be affected by an outlier signal if at least one keyword generated a positive signal). In addition, web-based surveillance performance was assessed separately for the three investigated regions because behaviours in online searches are expected to vary with the intensity of the epidemic and social patterns (58).

### 2.5. Sensitivity analyses

Sensitivity analyses were performed in addition to the primary analyses to assess the robustness of the results. First, we repeated the EWMA procedure using a less sensitive value of λ of 0.10 compared to 0.05 in the main analysis. Second, because the rule for generating an alarm are arbitrary, different and more stringent rules were also considered. These included generating an alarm signal only when the five keywords were taken together, and outliers could occur from: (i) at least two consecutive signals from at least one keyword, (ii) at least three consecutive signals from at least one keyword, and (iii) at least two keywords. Third, we verified whether Twitter posts might be used instead of Google search in the Italian setting (17). Finally, to verify whether the use of “negative keywords” (that is, syndromic proxies likely to be independent of COVID-19) may falsely predict the occurrence of a SARS-CoV-2 epidemic wave, negative keywords such as “cystitis,” “dizziness,” “fainting,” “tremor,” and “hallucinations” were used to recalculate the PPV and NPV. As signals generated from Lombardy were expected to be more stable than those from other regions, sensitivity analyses were performed using only data from Lombardy.

## 3. Results

From 1 March 2020 to 31 December 2021, 1,254,628, 147,085, and 358,740 confirmed cases of SARS-CoV-2 were ascertained in Lombardy, Marche, and Sicily, respectively, and the corresponding incidence rates were 12.1, 9.5, and 7.2 infections per 1,000 person-weeks.

Results from the ARMA(1,1) model are presented in the Supplementary material.

**Figure 1:**
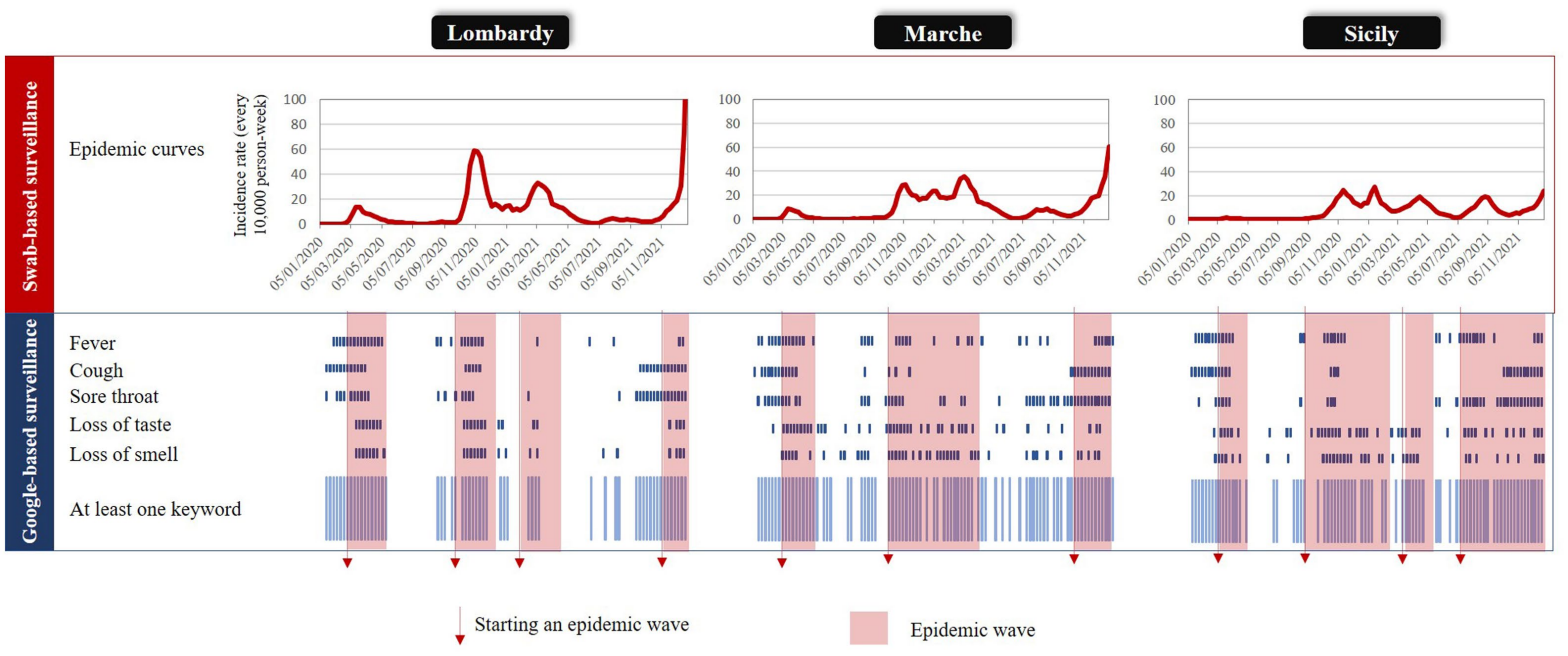
Comparing time-periods (weeks) affected by an epidemic wave and those affected by an outlier signal (from both specific keywords, and at least one keyword) in three Italian regions: Lombardy, Marche, and Sicily, 2020–2021.

Figure 1 compares the regional trends in SARS-CoV-2 infection rates observed from the swab-based surveillance system with weekly outliers generated from specific keywords and at least one keyword. According to our criteria, four, three, and four epidemic waves were ascertained in Lombardy, Marche, and Sicily, respectively. Although the corresponding rates had a progressively decreasing gradient from Lombardy to Marche to Sicily, the duration of the overall period affected by the epidemic excess was reversed. Of the 104 weeks of interest, 41 (39%), 48 (46%), and 68 (65%) respectively were affected by epidemic waves. Periods affected by outlier signals were heterogeneous between keywords and regions. Among keywords that were less often interested by outliers, “loss of taste” and “loss of smell” in Lombardy and “cough” in Sicily generated 23, 25, and 27 signals, respectively. In contrast, among keywords that were more often affected by outliers, “loss of smell” and “loss of taste” in Marche and “sore throat” in Sicily generated 48, 44, and 45 signals, respectively. Finally, the number of weeks affected by a signal generated by at least a keyword among the “free from epidemics” subperiods was 28 (44%) in Lombardy, 37 (66%) in Marche, and 18 (50%) in Sicily; of which, 82, 49, and 89% referred to the 8 weeks preceding the beginning of the epidemic wave, respectively.

**Figure 2:**
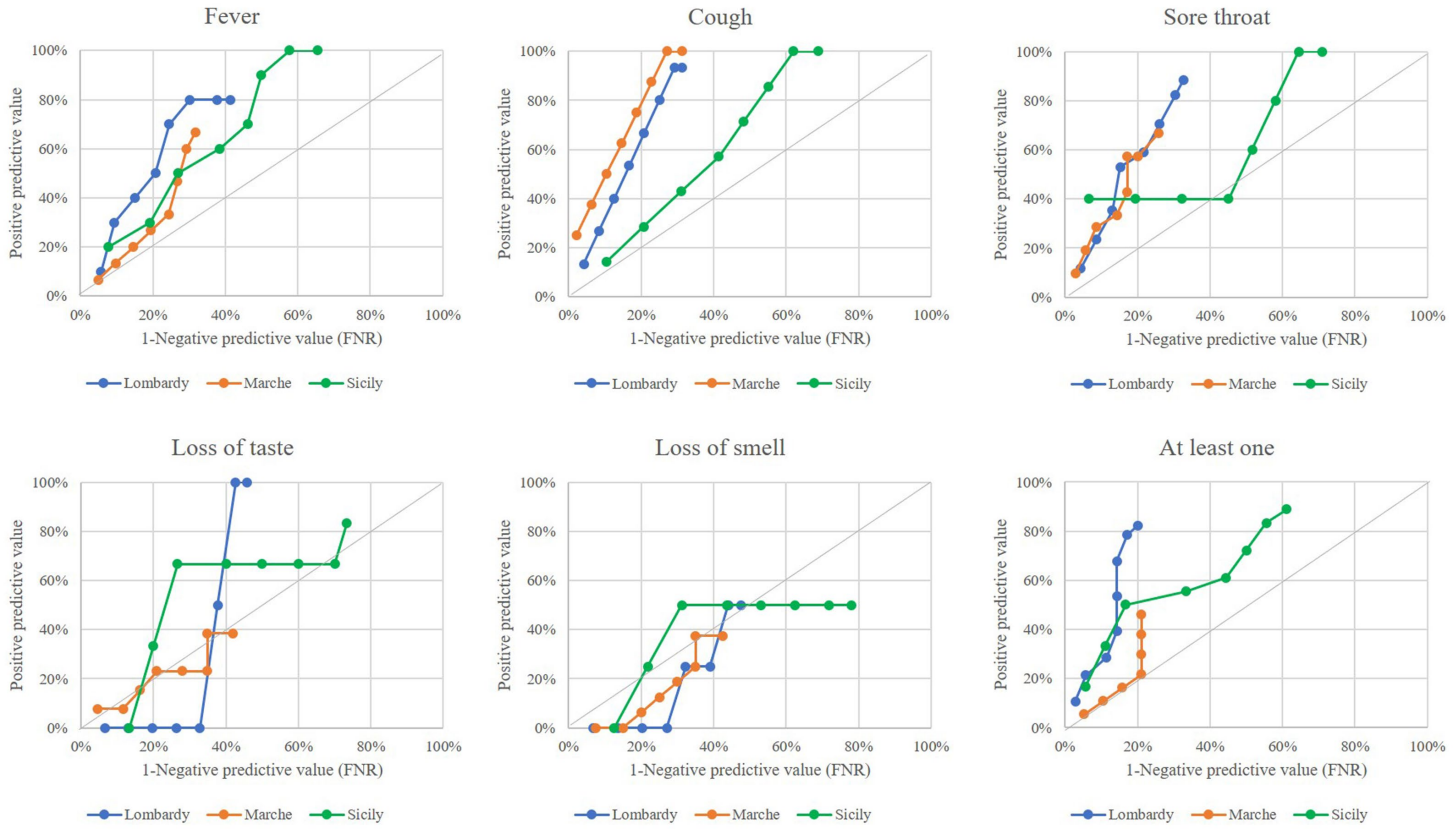
Comparing performance of outlier signals (from both specific keywords, and at least one keyword) for early onset of COVID-19 epidemic wave by varying the time-lag from outlier onset until the starting the epidemic wave from 1 to 8 weeks. Italian regions of Lombardy, Marche, and Sicily, 2020–2021.

The performance of outlier signals in anticipating the onset of an epidemic wave for each region is shown in Figure 2. Performance profiles were heterogeneous between keywords and between regions. The curves were almost always in the upper left hemi-quadrant for “cough,” “fever,” and “sore throat,” while they were almost always along the quadrant’s bisector for “loss of smell” and “loss of taste.” This suggests that outliers generated from the first three keywords, but not those from the last ones, were able to anticipate the onset of an epidemic wave. Using all keywords, Lombardy showed a better profile than Sicily and even more than Marche. Lombardy had a PPV of 80%, indicating that the onset of an epidemic wave may be detected 7–8 weeks before that from the swab-based surveillance system, with an FNR of 20%. In addition, the PPV in Sicily was 80%, with the FNR value of about 60%. Finally, the PPV in the Marches did not exceed 50%.

**Figure 3:**
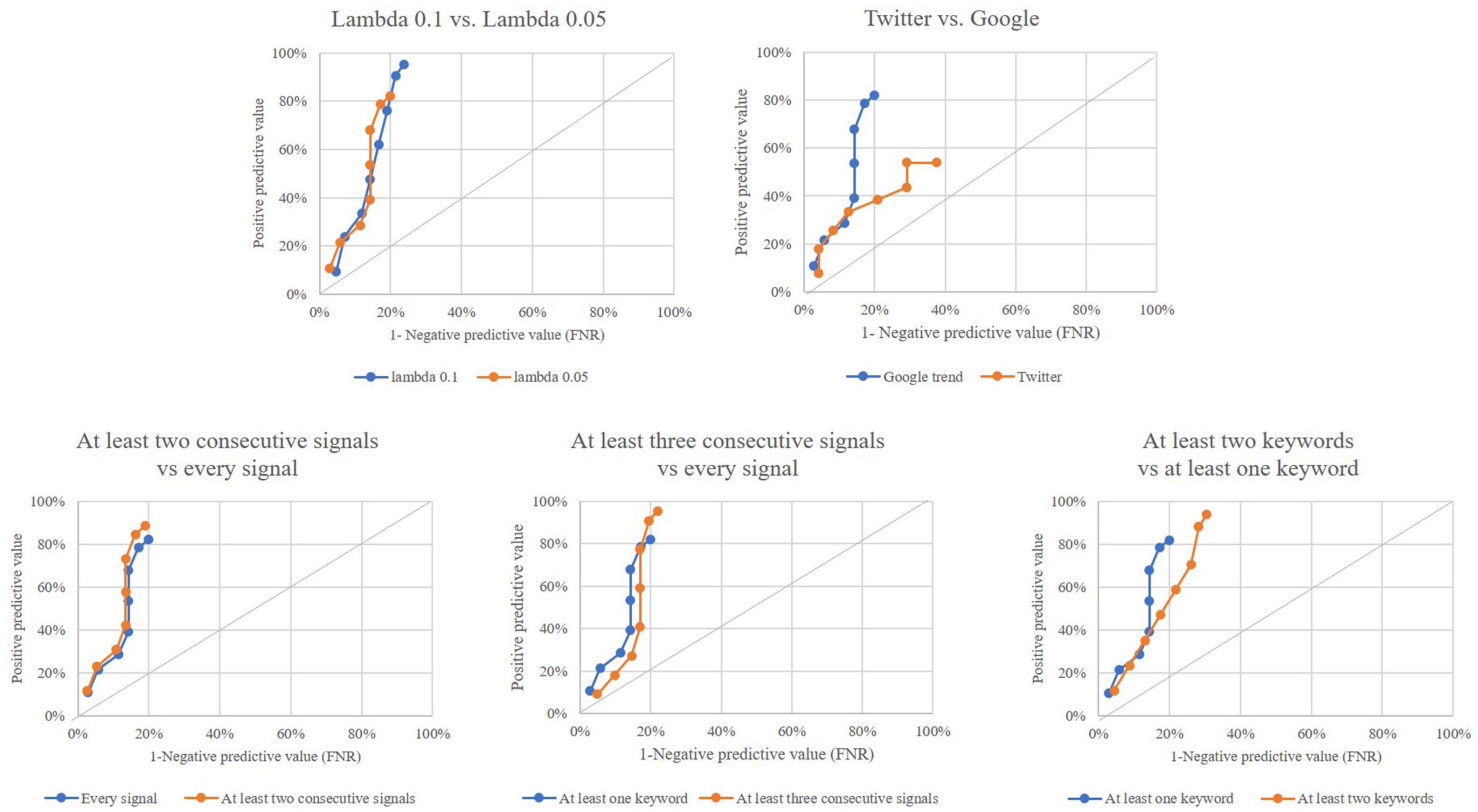
Performance of outlier signals (at least one keyword among those considered) for early detection of a COVID-19 epidemic wave by using (i) ʎ value of 0.10, instead of ʎ = 0.05 (top left box), (ii) twitter posts, instead of Google search (top right box), and (iii) more stringent rules for generating an alarm from individual outliers (bottom boxes). Italian region of Lombardy, 2020–2021.

Figure 3 shows that model performance did not change substantially by: (i) using a *λ* value of 0.10 for modelling the EWMA chart instead of 0.05 as in the main analysis or (ii) requiring more than one consecutive outlier to generate an alarm rather than using only one outlier as in the main analysis. Conversely, model performance was poor when: (i) using Twitter posts instead of Google searches as in the main analysis or (ii) requiring more than one keyword to generate outliers, rather than only one keyword as in the main analysis.

**Figure 4:**
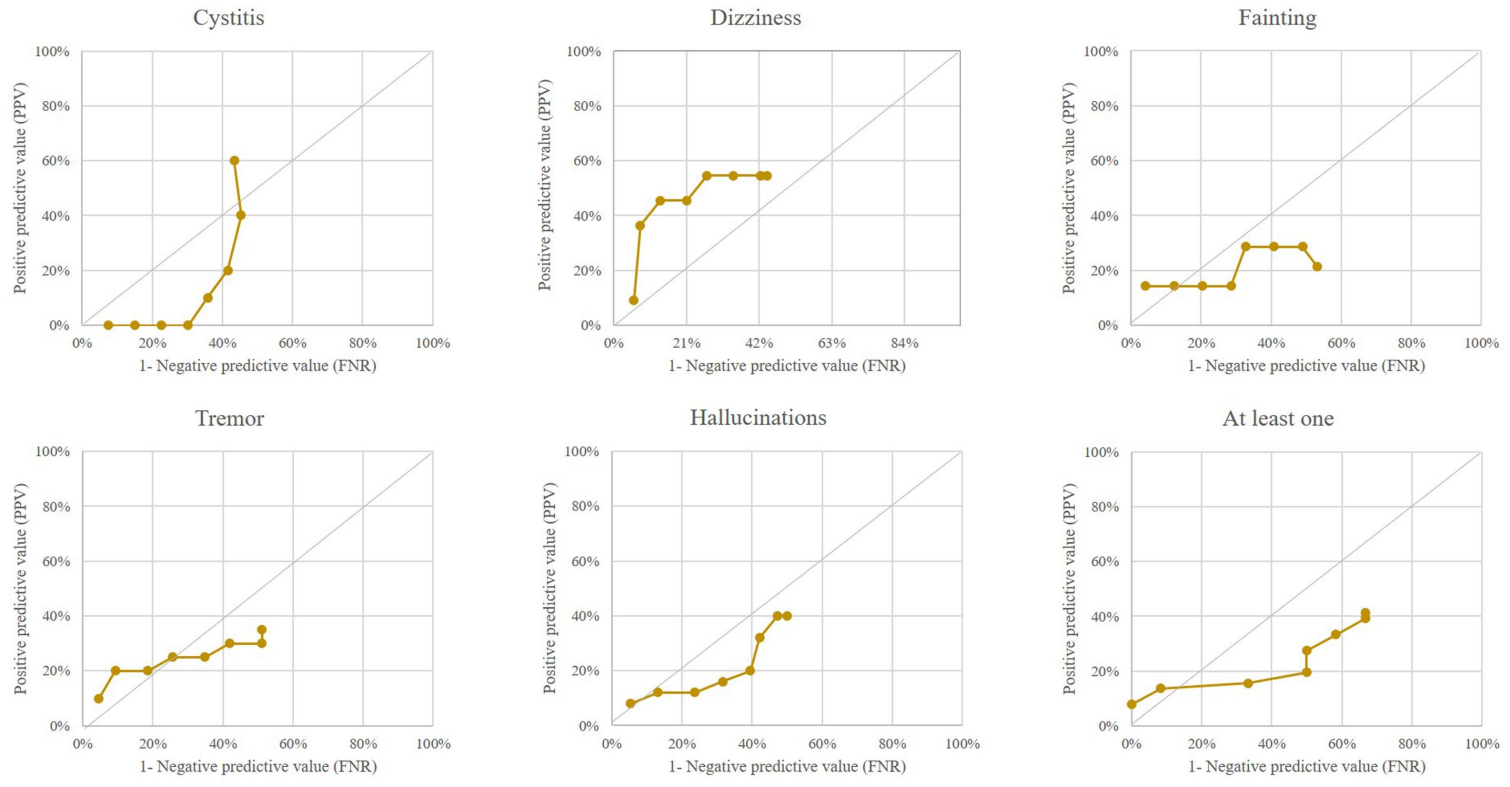
Performance of outlier signals from five “negative” keywords (and from at least one keyword among the five negative ones) for early detection of a COVID-19 epidemic wave. Italian region of Lombardy, 2020–2021.

Finally, Figure 4 shows that “negative keywords,” when considered individually (except for “dizziness”) or together, did not predict the occurrence of a SARS-CoV-2 epidemic wave early.

## 4. Discussion

We aimed to understand the potential of Google searches as early warning systems for the COVID-19 pandemic in Italy. Time-series of Google search on selected syndromic symptoms were compared with SARS-CoV-2 infection incidence rates based on nasopharyngeal swabs (official cases). Google-based outliers of the five investigated syndromic symptoms mainly occurred during epidemic waves. This is not surprising and confirms that flu-like syndromic symptoms, such as the investigated keywords, are mainly searched during periods of high viral spread. However, we aimed to verify whether the occurrence of Google-based outliers can detect outbreaks early with respect to the swab-based surveillance system. We found that keywords such as “fever” (consistently in the three investigated regions), and “cough” and “sore throat” (in Lombardy and Marche) showed good early detection ability. In contrast, “loss of smell” and “loss of taste” did not show similar abilities. Notably, by considering the five keywords, where the alarm is triggered by the occurrence of at least one outlier, over 80% of true epidemic waves were detected up to 7–8 weeks before their occurrence; however, 20% of the signals generated were false alarms. Unfortunately, these performances were achieved only in Lombardy, where citizens have the highest access to home Internet compared to other regions in Italy. Specifically, in 2021, 84.1% of housholds residing in Lombardy had Internet access from home, compared with 80.6 and 74.7% of housholds residing in Marche and Sicily, respectively (44). In addition, performances were not much less promising using Twitter posts rather than Google searches, suggesting that in Italy, especially Northern Italy, the Twitter spread is insufficient for syndromic surveillance. These findings suggest that web-based data sources, particularly Google Trends, may be a promising source for syndromic surveillance for early detection of flu-like epidemic spread compared to other more conventional sources. However, this is particularly true when the availability of internet access and systematic use of web-based platforms are widespread. The system fails in areas or regions with limited availability and use of the Internet.

Infodemiology metrics have been widely investigated during the current COVID-19 pandemic (3, 59, 60). While some studies employed fewer specific symptoms [e.g., fever, dry cough, fatigue, nasal congestion, and dyspnoea (39)], strong correlations have been reported with the more pathognomonic symptoms (e.g., anosmia and dysgeusia) (50). Our results contradicted these findings, as smell loss and loss of taste had the lowest predictive values for early detection of an epidemic wave compared to all non-pathognomonic symptoms. However, as our use of a control chart tool allows incorporating time-trend (progressively increasing use of the Internet) and seasonality (inherent to flu and cold seasons), we suspect that outliers generated by non-specific symptoms should be revaluated because they are early tracers of the onset of an epidemic wave. Conversely, we found that pathognomonic symptoms were concurrently correlated with epidemic waves but did not predict them early. Due to the high cultural, social, and behavioural potential related to this field, these findings likely reflect the Italian (and perhaps Mediterranean) setting that must be considered when designing the syndromic surveillance system. In general, surveillance based on Google Trends is limited by the influence of mass media communications as a possible effect of internet user behaviour (3). Thus, the generalizability of its utility in syndromic surveillance in space and time is questionable.

Some additional issues deserve to be addressed first, as several preventive public health measures have been taken worldwide, including Italy, to limit the spread of SARS-CoV-2, (i.e., hand hygiene and the use of masks, travel restrictions, social distance actions such as closing schools and workplaces, case and contact tracing, quarantine and isolation), the spread of other respiratory viruses, which occurs mainly by contact and drip, has also been contained (61–63). Notably, in just 1 month of the 2019–2020 winter season, SARS-CoV-2 became the most prevalent respiratory virus in northern Italy (64). This allows us to speculate that Google searches for infectious-respiratory symptoms are more likely to be related to Covid-19 than to other respiratory viruses.

Second, cumulative sum (CUSUM) chart models have been extensively used for syndromic surveillance systems worldwide (65–67). Although our study was not designed for comparing chart models, according with our findings EWMA, we observed that the EWMA seems to identify earlier and more accurately alerts generated by an abnormal increase in the weekly volume of Google Trends searches on respiratory syndrome-related keywords (Supplementary Figure S1). This is not surprising because, with respect to CUSUM, EWMA has been reported to be robust to deviation from normality (68, 69) and showed particular skills for detecting small shifts in the mean of a process (70). On the other hand, EWMA chart had been found to be more prone to false alarm counts with respect to other approaches (71), and this potential weakness should be careful considered in a surveillance syndromic system.

Second, we used official data on the positivity of nasopharyngeal swabs as a proxy for the gold standard, which is the weekly count of SARS-CoV-2 infections. However, it should be considered that the proxy systematically underestimates the gold standard and that the underestimation changes over time. For example, only a small proportion of infected individuals were detected at the beginning of the epidemic shock (a period when we were not ready to face the emergency). Notably, the syndromic surveillance system we proposed works better when tracing based on nasopharyngeal swabs is inadequate.

Our findings represent a useful and promising starting point and suggest that some improvements are needed before the system can be applied systematically as an early warning method. Our best result estimates that 80% of the emerging outbreaks could be identified early by the system. However, a high number of false signals would also be generated (about 20%). Although the number of false positives can be considered acceptable depending on the type of public health intervention following the generation of an alarm (e.g., adoption of restrictive measures, localised diagnostic testing, and alerting hospitals and general practitioners), our findings are insufficient to recommend systematic applications. However, the high number of false signals could be partially explained by the uncertainty in defining confirmed outbreaks. It is possible that some outbreaks that occurred were not detected by standard surveillance (or did not match the confirmed outbreak definition used) but were detected by monitoring the use of health services. This might have generated a conservative estimate of the system performance (72).

In conclusion, using Google Trends to identify control chart-based outliers for non-pathognomonic symptoms such as fever, cough, and sore throat has high predictive power for anticipating COVID-19 epidemic waves 7–8 weeks ahead of the official reports in Lombardy. If combined with other syndromic sources like those of data from healthcare utilisation (8) and emergency visits (7), data from Google Trends searches may serve as a useful infodemiological tool for anticipating an impending outbreak, which can provide valuable buffer time to allocate the necessary supplies and personnel to hospitals expecting a surge in COVID-19 patients. Upon verification by prospective research comparing model performance in different regions of Italy, public health organisations are encouraged to take advantage of this free forecasting system to anticipate and effectively manage COVID-19 outbreaks throughout Italy.

## Data availability statement

Publicly available datasets were analyzed in this study. This data can be found here: Google trend data: https://trends.google.it/trends/?geo=IT; Covid-19 confirmed cases data: https://github.com/pcm-dpc/COVID-19/tree/master/dati-regioni.

## Author contributions

GC was involved in study conception. GC and GP contributed to the study design and methodology. GP, YC, AB, and LR analyzed the data and had full access to all the data in the study and take responsibility for the integrity of the data and the accuracy of the data analysis. GC and GP prepared the draft manuscript. YC, AB, SV, LR, VM, GB, MM, OL, DC, FM, and AG contributed to and reviewed the manuscript. All authors contributed to the article and approved the submitted version.

## Funding

This study was funded by Polis Lombardia (project unique identification code: H45H20000400002). The funding source had no role in the design and conduct of the study; collection, management, analysis, and interpretation of the data; preparation, review, or approval of the manuscript; and decision to submit the manuscript for publication.

## Conflict of interest

GC received research support from the European Community (EC), the Italian Agency of Drug (AIFA), and the Italian Ministry for University and Research (MIUR). He took part to a variety of projects that were funded by pharmaceutical companies (i.e., Novartis, GSK, Roche, AMGEN and BMS). He also received honoraria as member of Advisory Board from Roche.

The remaining authors declare that the research was conducted in the absence of any commercial or financial relationships that could be construed as a potential conflict of interest.

## Publisher’s note

All claims expressed in this article are solely those of the authors and do not necessarily represent those of their affiliated organizations, or those of the publisher, the editors and the reviewers. Any product that may be evaluated in this article, or claim that may be made by its manufacturer, is not guaranteed or endorsed by the publisher.

## Supplementary material

The Supplementary material for this article can be found online at: https://www.frontiersin.org/articles/10.3389/fpubh.2023.1141688/full#supplementary-material
