# Supplementary material for: Web-based surveillance of respiratory infection outbreaks: retrospective analysis of Italian COVID-19 epidemic waves using Google Trends
Source: Front Public Health. 2023 May 18;11:1141688. doi: 10.3389/fpubh.2023.1141688 (PMC10233021; doi:10.3389/fpubh.2023.1141688)

Supplementary Material

Web-based surveillance of respiratory infection outbreaks: Retrospective analysis of Italian COVID-19 epidemic waves using Google Trends

Gloria Porcu, Yuxi Chen, Andrea Bonaugurio, Simone Villa, Leonardo Riva, Vincenzina Messina, Giorgio Bagarella, Mauro Maistrello, Olivia Leoni, Danilo Cereda, Fulvio Matone, Andrea Gori, Giovanni Corrao*

*** Correspondence:** Prof. Giovanni Corrao, Dipartimento di Statistica e Metodi Quantitativi, Università degli Studi di Milano-Bicocca, Via Bicocca degli Arcimboldi, 8, Edificio U7, 20126 Milano, Italy. Tel.: +39.02.64485854; E-mail: giovanni.corrao@unimib.it

|  | **Fever** | | | **Cough** | | | **Sore throat** | | | **Loss of taste** | | | **Loss of smell** | | |
| --- | --- | --- | --- | --- | --- | --- | --- | --- | --- | --- | --- | --- | --- | --- | --- |
| Parameter | Estimate | SE | Pr > \|t\| | Estimate | SE | Pr > \|t\| | Estimate | SE | Pr > \|t\| | Estimate | SE | Pr > \|t\| | Estimate | SE | Pr > \|t\| |
| Intercept | 25.593 | 2.88879 | <.0001 | 31.9869 | 4.14526 | <.0001 | 23.4112 | 3.55423 | <.0001 | 1.48895 | 0.52633 | 0.0054 | 0.95666 | 0.58681 | 0.1053 |
| January (M1) | -1.679 | 4.51574 | 0.7106 | 6.84268 | 7.23317 | 0.3458 | 2.01502 | 6.62081 | 0.7613 | -1.79526 | 1.00327 | 0.0757 | -0.92259 | 1.13056 | 0.4159 |
| February (M2) | -4.8313 | 4.41472 | 0.2757 | 2.28881 | 7.00033 | 0.7442 | 0.22786 | 6.40028 | 0.9717 | -1.98613 | 0.96944 | 0.0424 | -0.20899 | 1.09222 | 0.8485 |
| March (M3) | -6.74 | 4.06295 | 0.0994 | -0.2924 | 6.42743 | 0.9638 | 1.99961 | 5.83998 | 0.7326 | -1.25483 | 0.87408 | 0.1533 | -0.01855 | 0.98156 | 0.9849 |
| April (M4) | -6.2823 | 3.55166 | 0.0791 | -0.66841 | 5.53268 | 0.904 | 1.80605 | 4.94348 | 0.7154 | -0.85001 | 0.72427 | 0.2425 | 0.2868 | 0.80692 | 0.7228 |
| May (M5) | -2.6952 | 2.95367 | 0.3631 | 4.76675 | 4.51471 | 0.2929 | 4.87983 | 3.81639 | 0.2031 | -0.31102 | 0.54878 | 0.5718 | 0.66015 | 0.60399 | 0.2763 |
| June (M6) | -1.3354 | 2.12573 | 0.5309 | 2.47344 | 3.27958 | 0.452 | 1.76598 | 2.65438 | 0.5069 | 0.17688 | 0.36486 | 0.6286 | 0.64597 | 0.39522 | 0.1044 |
| July | 0 | ref | | 0 | ref | | 0 | ref | | 0 | ref | | 0 | ref | |
| August (M7) | -0.9942 | 2.15334 | 0.645 | 0.39469 | 3.27951 | 0.9044 | 2.07339 | 2.64014 | 0.4336 | 0.09461 | 0.36626 | 0.7965 | -0.07723 | 0.39433 | 0.8450 |
| September (M8) | -2.4862 | 2.92357 | 0.3966 | -1.46818 | 4.52271 | 0.746 | 4.7041 | 3.78385 | 0.2159 | -0.23182 | 0.55109 | 0.6747 | -0.48013 | 0.60714 | 0.4304 |
| October (M9) | -3.5935 | 3.5698 | 0.3158 | 3.4395 | 5.61888 | 0.5414 | 4.44799 | 4.97059 | 0.3724 | -1.16346 | 0.74351 | 0.1199 | -1.36791 | 0.82884 | 0.1011 |
| November (M10) | -3.8573 | 4.09271 | 0.3476 | -2.50879 | 6.4971 | 0.7 | 1.9092 | 5.78333 | 0.7418 | -1.88901 | 0.88463 | 0.0345 | -1.1904 | 0.99368 | 0.2330 |
| December (M11) | -1.6468 | 4.47156 | 0.7132 | 6.10608 | 7.10085 | 0.3913 | 8.72272 | 6.33578 | 0.1708 | -1.97508 | 0.97335 | 0.0443 | -1.21471 | 1.0968 | 0.2700 |
| Sine (sin) | 2.23774 | 2.39697 | 0.3521 | 0.21985 | 3.67088 | 0.9523 | 1.77545 | 3.27693 | 0.5888 | 0.23561 | 0.4886 | 0.6304 | -0.42814 | 0.54624 | 0.4345 |
| Cosine (cos) | 6.53612 | 2.32016 | 0.0055 | 16.3327 | 3.5706 | <.0001 | 6.64249 | 3.20615 | 0.0401 | 1.26474 | 0.49281 | 0.0113 | 0.78123 | 0.55681 | 0.1628 |
| Trend variable (t) | -0.0024 | 0.00262 | 0.3564 | -0.0001 | 0.00269 | 0.9696 | -0.0015 | 0.00161 | 0.3541 | -0.00015 | 0.00019 | 0.4273 | 9.1E-05 | 0.00018 | 0.6198 |
| MA1,1 | -0.06279 | 0.14694 | 0.6698 | -0.33012 | 0.15301 | 0.0327 | -0.1418 | 0.30826 | 0.6462 | 0.47101 | 1.45922 | 0.7473 | -0.36751 | 0.51385 | 0.4757 |
| AR1,1 | 0.64079 | 0.12414 | <.0001 | 0.31116 | 0.16116 | 0.0555 | 0.17399 | 0.31386 | 0.5802 | 0.51132 | 1.42138 | 0.7196 | -0.49444 | 0.47814 | 0.3029 |

**Supplementary Table 1.** Parameters of regression model with ARMA (1,1) errors.

**Supplementary Figure 1.** Signal alarms generated by CUSUM and EWMA chart control models in Lombardy region.


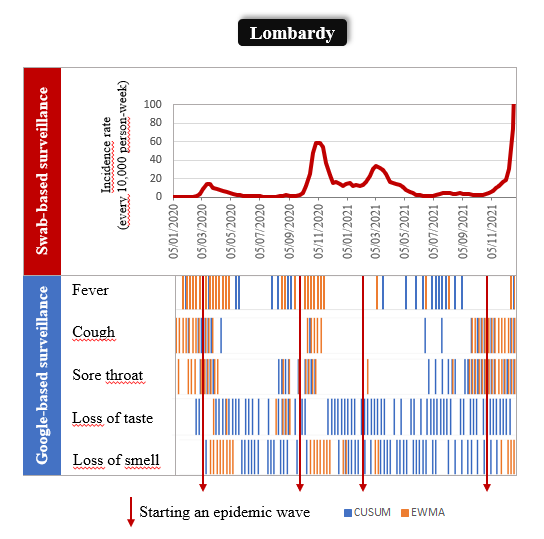

Supplement: Supplementary file 1 [file Data_Sheet_1.docx]
